# Addressing quality and safety in anatomic pathology in low- and middle-income countries

**DOI:** 10.3389/fmed.2022.1060179

**Published:** 2022-12-22

**Authors:** Stephen M. Smith, Amrik Eadara, Vinita Parkash

**Affiliations:** ^1^Department of Laboratory Medicine & Pathobiology, University Health Network, Toronto, ON, Canada; ^2^Brown University, Providence, RI, United States; ^3^Department of Pathology, Yale School of Medicine, Yale University, New Haven, CT, United States

**Keywords:** quality, anatomic pathology, LMIC (low- and middle-income countries), Africa, patient safety, quality assurance

## Abstract

The World Health Organization (WHO) has created a sustainable development goal of reducing preventable mortality from cancer in low- and middle-income countries (LMICs) by 30% by 2030. Central to achieving this goal is the creation and maintenance of quality anatomic pathology services (APS). Within the last decade, quality assurance programs and patient safety measures have become a major focus of research for upper middle- and high-income countries (UMHICs), which has led to marked documented improvement in the quality of services provided by laboratories, as well as a decrease in patient safety events. We propose that as APS are developed in LMICs, the lessons learned by UMHICs are necessary to incorporate to produce quality and safe services toward obtaining the aforementioned goal. Furthermore, data suggests that Quality Improvement work requires change at the macrosystems and microsystems levels to achieve these goals. Here, we propose five “microsystems” strategies for professional organizations, healthcare institutions in LMICs and UMHICs that would accelerate quality improvement programs/systems implementation in APS in LMICs.

## 1. Introduction

Cancer is the second leading cause of death from non-communicable diseases in low- and middle-income countries (LMICs). It is estimated that 75% of cancer deaths worldwide will occur in LMICs by 2030 (1). Achieving the World Health Organization (WHO)/World Bank sustainable development goal (SDG) of reducing preventable mortality from cancer in LMICs by 30% by 2030, requires that LMICs have reliable, consistent attention to a delivery structure for Anatomic Pathology Services (APS) (2).

Anatomic diagnosis is central to the diagnosis and treatment of malignancy at the individual patient level. Histopathologic evaluation assigns subtype, grade and microscopic stage, and identifies secondary prognostic features and molecular signals that predict prognosis and the choice of therapy for cancer. APS also play a critical role at the public health level for the prevention of cancer. Cervical cancer, for example, has been well-controlled in the upper middle- and high-income countries (UMHICs) through pap smears—the single most successful prevention strategy for cancer, but which has defied implementation in LMICs (3). Cervical cancer prevention in LMICs is also limited by the fact that human papilloma virus (HPV) testing (and vaccines), now commonplace in North America, is too expensive for implementation in LMICs (4). Lastly, APS are critical to providing data for cancer registries and informing on the evolving epidemiologic patterns of cancer in a country to direct policy and resource allocation.

Pathology and Laboratory Medicine services, the broader group within which APS is situated, face substantial implementation challenges in LMICs (5). Wilson et al. identify four critical issues: (1) insufficient human capacity; (2) inadequate infrastructure; (3) inadequate education and training; and (4) inadequate quality, standards and accreditation. To date, laboratory services improvement efforts have necessarily focused on policies and processes of developing capacity and infrastructure at a macrosystems level (6).

However, as has become clear in the last decade, quality is essential and as much imperative as access to improve outcomes; poor quality is a bigger barrier to reducing mortality than access (7–9). Furthermore, evidence suggests that quality of care in extreme low resource and low resource settings, is challenged not, as was previously believed, by overwork, but by underwork, large know-do gaps and suboptimal context-level effective management (10). In addition, determinants of healthcare professional practice (organizational readiness, culture, and behavior of professionals) are critical to implementation success, and thus form integral components of implementation frameworks such as the Consolidated Framework for Implementation Research (CFIR) (11–13). Low resource organizational microsystems face similar organizational-cultural behaviors that create inefficiencies and result in ineffective and inconsistent processes which limit healthcare delivery in UMHICs (10, 14). Professional-cultural behaviors and resistance to change are among the greatest barriers to quality outcomes (14). This evidence suggests that delivering and implementing high quality outcomes requires a combined approach with efforts that are top-down, macro-mesosystems level and policy-regulatory, and microsystems-level, bottom-up and frontline organizational-institutional (10). Thus, even as countries work toward creating policy, regulatory, and infrastructure to improve access to APS, we suggest that microsystems “quality improvement and cultural change” work at the organizational levels needs to begin in LMICs to ensure that capacity and infrastructure investment deliver results (15). UMHICs need to partner with LMICs to jumpstart and accelerate this movement.

Effort has started with respect to microsystems quality improvement for laboratory medicine services, especially under the auspices of Strengthening Laboratory Management Toward Accreditation (SLMTA); it is primarily directed toward clinical laboratory services (16). However, APS requires a separate and particular consideration because of its positioning in diagnosis. The practice of diagnostic surgical pathology is higher risk—it is often the final word on cancer—one that overrules virtually all other tests; the tissue sample is an irretrievable, irreplaceable specimen. Moreover, APS professionals (pathology assistants, histotechnologists, cytotechnologists) and surgical pathologists require different training and skillset development, for a more manual laboratory structure, that requires a more hands-on-quality improvement methodologic knowledge base, compared to clinical laboratory medicine professionals.

Here, we propose five strategies to start a bottom up, microsystems level movement for quality improvement in LMICs, assisted by partners including colleagues, professional organizations and healthcare institutions in UMHICs. Implicit in these considerations are potential lessons for UMHICs to address waste, low-value care and disparities of care that plague some UMHICs.

## 2. Setting the expectations of diagnostic quality and accuracy begins with education: Education in quality and safety should be incorporated into all levels of healthcare and pathology curricula in LMICs

Although UMHICs have made significant strides in achieving impressive health outcomes, this has come at the cost of significant waste, and disparate outcomes especially for low resource and vulnerable populations. It is estimated that 30% of healthcare costs in the US are a waste from low-value care; a significant amount from unnecessary, repeat and wasteful testing (17). It is also estimated that there is significant delay to universal uptake of evidence-based practice guidelines even in UMHICs (18). To address these weaknesses in the healthcare delivery structure, the US has committed to quality in health with its six pillars—safe, timely, efficient, effective, equitable and patient centered care. To achieve this goal of quality and safety (Q and S) in healthcare, the Accreditation Council for Graduate Medical Education (ACGME) now requires dedicated Q and S training be part of residency training in the United States (19). The goal of this training is producing practitioners who can evaluate their own unsupervised practice and institute quality improvement measures for lifelong learning and delivery of high-quality care. An important reason for instituting this change at the trainee level is that professional cultural change is critical for a safety culture mindset, and habits are hard to break.

We propose that all APS training at all levels (technical through professional) in LMICs incorporate and require basic training in quality and safety. This is critical to empower local pathologists and laboratorians to use Evidence to Decision (EtD) frameworks to assess and transform UMHIC-developed protocols to local settings (20). A local *de jure* standard that conforms to the highest level possible requires considerations of feasibility, cultural acceptance and balances benefits against harms (21). The blind adoption of specimen processing protocols from UMHICs, developed for patient populations where the primary presentation of cancer is early, where the harms-benefits equation differs, and where the choice of treatment interventions varies significantly will likely result in significant waste in an LMIC setting.

The SLMTA and Stepwise Laboratory Improvement Process Toward Accreditation (SLPTA) quality improvement programs have achieved significant gains in advancing quality and safety in clinical laboratories in LMICs (16, 22). However, APS-specific Q and S protocols are not part of this process. Q and S training is particularly important in APS as it is a more manual, less automated, process. Histochemical and immunohistochemical tests are laboratory-developed and require constant tweaking, monitoring and modification, using a structured quality improvement approach, to achieve optimal performance.

Basic “general medicine” Q and S training is available from various sources. We favor the Q and S modules from Institute of Healthcare Improvement, which offers a basic certification in Q and S for free for healthcare providers (23). While not APS specific, training in the basic principles of quality and safety ensures that medical professionals develop a mindset that is attentive to quality and begins the journey toward organizational and professional culture change, so that organizations achieve change readiness to reap the rewards of infrastructure investment. Data shows that even in low resource settings, a culture of safety is critical to optimizing outcomes (24, 25). Furthermore, the Model for Improvement (Plan-Do-Study-Act) is a model that is applicable at the microsystems level and allows for incremental quality improvement even in resource-limited settings (26). Every pathology department should train and designate an individual responsible for departmental quality and patient safety. Education in the basic principles of quality and safety will allow individuals in existing structures to not just optimize outcomes but identify and develop quality metrics that are context specific.

Anatomic Pathology Q and S education modules are in development as part of the Open Pathology Education Network (OPEN) project, which aims to develop a digital pathology enabled virtual curriculum to improve the global pathology workforce, using the DPA-DAPA (Digital Pathology Association and Digital Anatomic Pathology Academy) resources (27–30).

## 3. Develop resource-stratified external quality assurance and quality improvement collaboratives for LMIC regions

Laboratories in similar settings face similar challenges and can benefit from each other. Project ECHO (Extension for Community Health Outcomes), from the University of New Mexico, is an “all teach, all learn” education and care management model, that “moves knowledge, not people” (31). It is a model based in virtually connecting healthcare professionals to experts to address care-management problems and improve patient outcomes. The model has been successful in providing consultant and expert support to peripherally located generalist clinicians, remote oversight of tumor boards, and support to teaching hospitals in LMICs. The model has been successfully leveraged in at least two settings with respect to laboratory medicine and APS. In one model, pathologists participate in providing tumor-board support to International Gynecological Cancer Society supported gynecological oncology training programs in LMICs (32). The Division of Laboratory Systems, Centers for Disease Control and Prevention (CDC) successfully piloted a laboratory community of practice (CoP) on diagnostic excellence using an adaptation of the clinician-based ECHO Model. The project aims were to (1) connect laboratory professionals, clinicians, and subject matter experts in laboratory medicine, (2) use case-based learning to educate and train laboratory and healthcare professionals, and (3) examine cross-cutting issues in clinical and anatomic pathology (33). Serendipitously, this project was ongoing during the recent COVID-19 pandemic and facilitated transfer of knowledge and lessons learned from frontline states to other states and allowed them to prepare somewhat for the onslaught of the coming pandemic (34).

Extension for Community Health Outcomes-like models, combined with telepathology, are high potential for APS-efforts for diagnostic quality improvement and education, especially as the COVID pandemic has accelerated the adoption of digital diagnostic APS in UMHICs (35). To date, telepathology efforts have been used to provide diagnostic support from a UMHIC collaborator to an LMIC site (36). A small External Quality Assurance (EQA) in diagnosis for APS for LMICs (Ghana and Nigeria) hosted by Leeds University is also reported (37) (the authors were unable to access the further data about this program). However, there is likely a need to develop LMIC context-specific EQAs as the use of EQAs in UMHICs may not necessarily improve implementation in LMICs.

Quality improvement collaboratives (QICs) aim to bring together institutions (laboratories) to implement evidence-based quality improvement initiatives. These have been used in a variety of specialties, often across geographic borders. They have been instrumental in improving infectious disease care (particularly tuberculosis and human immunodeficiency virus care) (36). The existing structures are now being leveraged to expand the initiatives to non-infectious diseases with the hopes of accelerating the time to achieving the non-infectious disease SDGs (38). To our knowledge, QICs have not been used in APS.

One of the authors (VP) is attempting start one such initiative for extreme low resource settings where even low-resource models are untenable (39). She is among a small group of pathologists who is participating in a pilot to provide remote pathology services at ECWA Hospital, Egbe, Nigeria. The program is modeled on a successful grass-roots effort at the Mbingo Baptist Hospital Pathology program in Cameroon. A relative low-cost ($10,000 USD) manual telepathology set up from Microvisoneer^TM1^ allows pathologists in the US to provide diagnostic services; a similar project has been started in Djibouti (40).

## 4. Development of resource-stratified stage-based guidelines for pathologic assessment and reporting of malignancy should be a priority for achieving an appropriate standard of care in LMICs

Pathologic evaluation protocols that are high quality and provide accurate diagnoses and information allow for timely and appropriate intervention. With rapid advancements in technology, molecular characterization of tumors has become essential to subtype tumors and to allow for targeted therapies in UMHICs. However, this diagnostic standard has the potential to increase the gap in diagnostic accuracy between UMHICs and LMICs, because the diagnostic criterion is the molecular signature (e.g., the diagnosis of partial mole requires the demonstration of diandric triploidy). While this progress should not be curtailed or decelerated in UMHICs, especially as it will eventually benefit patients even in LMICs, there is need to develop evidence-based protocols that are resource-stratified.

The key standard is an evidence-based, stage and resource-stratified guideline for pathologic cancer assessment and reporting. This is needed both at the gross and diagnostic level as a standard of care that is insensitive to implementation factors will necessarily fail.

Grossing protocols developed in UMHICs where malignancy presents early are likely to be wasteful in LMICs. The two most important predictors of outcome for cancer are histologic type and stage. Considering that cancers in LMICs not infrequently present at advanced stages (secondary to lack of screening availability more commonly seen in UMHICs), the extensive blocking-grossing protocols of UMHICs add cost without value in LMICs. Stage-stratified grossing protocols are one possible solution and may help reduce waste even in UMHICs, as current grossing protocols in UMHICs are independent of stage parameters. The incremental value from expansive sectioning of the ovary and uterus, in the setting of widely metastatic peritoneal carcinoma is unclear. A more cost-effective strategy may be to submit 2–3 sections of the metastatic tumor for diagnosis. Similarly, detailed subtyping of tumors (determining serous carcinoma vs. high grade endometrioid carcinoma) may not be value-added, if no treatment differences exist in a resource-limited setting.

Clinical societies have already adopted this approach and have partnered with clinical domain experts in Sub-Saharan Africa to propose alternative pathologic evaluation guidelines for resource limited settings. However, at least in some cases, these are incongruous as they are created without knowledge of pathology processing (41). Thus NCCN guidelines suggest that ultrastaging may not be feasible in low-resource settings, but recommend reporting of isolated tumor cells and micrometastases, which necessarily require ultrastaging (42). Some ignore more simplistic and efficient strategies to obtain relevant information. The direct involvement of surgical pathologists in the creation of such guidelines is critical, especially from those with experience or expertise in resource-limited settings. Involvement of experts from developing countries (e.g., India) may be particularly beneficial as they may bridge the gap between extreme low resource and high resource settings and may offer expertise that has been lost in UMHICs. A challenge faced by one of the authors when participating in a cervical cancer grassroots effort in South India was that few cytologists or cytopathologists in the US felt competent to read routine pap smears; they had all transitioned to or only trained in liquid based cytology.

## 5. Academic institutions associated with UMHICs should expand affiliations to include pathology at outreach facilities in LMICs

Many academic medical centers in UMHICs have affiliate programs at universities in LMICs, to enrich educational experiences in infectious disease and low-resource primary-care rotations for trainees (43). This infrastructure can be leveraged to build bridges between pathology departments in UMHICs and LMICs. Indeed, two of the most successful UHMIC-LMIC pathology programs—one in Rwanda and the other in Malawi were developed by leveraging existing connections for infectious diseases (44, 45). This structure should also allow for exchange of knowledge for better diagnostic accuracy. In general, points for consideration would include:

1. Leverage telepathology to offer consultations and more to affiliates in LMICs.
2. Create structured bilateral exchange programs for short-term rotation for visiting pathologists and laboratory professionals between affiliate UMHIC and LMIC academic programs.
3. Develop mechanisms by which affiliate pathologists in LMICs can attend educational, clinical and Q and S conferences in UMHIC. COVID created pressure for educational programs in UMHICs to move to hybrid learning modules (45). This offers unprecedented opportunity to include trainees and pathologists in LMICs in educational offerings without added costs to either site. We hope that UMHICs will consider partnering with pathology programs in LMIC for improved education and training within existing structures. The development of collaborative educational efforts also enriches the educational experiences of trainees in UMHICs, giving them a perspective of global health issues. This also helps to develop relationships between trainees across disparate settings that could sustain through their careers and help strengthen bridges between pathology programs in LMICs and UMHICs.
4. An eventual goal maybe to develop primarily on-line training models for LMICs, especially for training laboratory professionals.

## 6. Engagement of the international medical graduate diaspora in UMHICs for the education of and development of LMIC pathology services

Pathology services in UMHICs are provided to significant extent by providers who have roots in LMICs. In 2022, 45% of entry level trainees in the US in pathology were international medical graduates (IMGs) (46); a significant plurality from LMICs. There is anecdotal evidence that many IMGs—particularly later in life, after achieving financial and child-rearing obligations— look to contribute to advancing healthcare in the LMIC country of their roots. This connection could be tapped and encouraged by institutions in UMHICs for collaborative efforts and support for LMICs.

Current reward structures in UMHIC academic institutions value publications in prestigious journals, typically of studies with molecular inquiry of disease, and presentations at UMHIC national meetings or at institutions, while discounting public health and educational efforts in LMICs for academic advancement. Service on certain types of committees in professional societies in UMHICs is also valued over involvement in less visible grass-roots efforts in low-resource settings. Recognition of work in LMICs as a value-added effort rather than simple volunteerism could incentivize greater involvement in LMIC work from IMGs in UMHICs. Furthermore, professional societies should look to this untapped resource for LMIC work as these individuals have cultural, language and context specific competencies to move LMIC programs forward more quickly.

## 7. Conclusion

In summary, improving the quality of APS in LMICs is essential to achieving SDG. While significant effort is still needed from countries at the policy and infrastructure levels, some changes, including education in quality improvement at the microsystems levels (institutional and organizational levels) within LMICs will allow for improved quality and optimization of existing diagnostic services and create organizational readiness and cultural change to optimize returns from infrastructure investments. These investments, while small, have the potential, to allow for adoption and adaptation of evidence-based models to local constraints and identify novel strategies for success in low resource settings.

Academic institutions in UMHICs need to invest in establishing foundational and sustainable educational and clinical frameworks to promote quality pathology services in LMICs. Changing incentive and advancement reward structures in UMHICs academic institutions can promote greater investment from the IMG diaspora in LMIC work. Professional organizations need to commit more toward these efforts and engage the IMG diaspora in pathology in LMIC work; the IMG diaspora offers a ready resource that remains untapped. These small but important changes have the potential to help the world meet SDG for cancer.

## Data availability statement

The original contributions presented in this study are included in the article/supplementary material, further inquiries can be directed to the corresponding author.

## Author contributions

VP, SS, and AE were involved in the creation, drafting, and revising of this manuscript. All authors agree with the manuscript as submitted herein.

## Footnotes

1
https://www.microvisioneer.com/
